# The cost of elective nodal coverage in prostate cancer: Late quality of life outcomes and dosimetric analysis with 0, 45 or 54 Gy to the pelvis

**DOI:** 10.1016/j.ctro.2022.06.008

**Published:** 2022-06-27

**Authors:** Garrett L. Jensen, Sameer G. Jhavar, Chul S Ha, Kendall P. Hammonds, Gregory P. Swanson

**Affiliations:** a Departments of Radiation Oncology, Baylor Scott & White Health, 2401 S. 31st St., Temple, TX 76508, USA; b Department of Radiation Oncology, UT Health San Antonio, 8300 Floyd Curl Dr., San Antonio, TX 78229, USA; c Departments of Biostatistics, Baylor Scott & White Health, 2401 S. 31st St., Temple, TX 76508, USA

**Keywords:** PLNRT, pelvic lymph node radiotherapy, RT, radiotherapy, PROMs, patient-reported outcome measures, QOL, quality of life, RP, radical prostatectomy, EPIC, Expanded Prostate Cancer Index Composite, ADT, androgen deprivation therapy, MID, minimally important difference, MVA, multivariate analysis, AUC, areas under the curve, PPV, positive predictive value, Elective nodal radiation, Patient reported outcome measures, Quality of life

## Abstract

**Purpose:**

Elective pelvic lymph node radiotherapy (PLNRT) in prostate cancer is often omitted from definitive (n = 267) and post prostatectomy (n = 160) radiotherapy (RT) due to concerns regarding toxicity and efficacy. Data comparing patient-reported outcome measures (PROMs) with or without PLNRT is limited. Our long-term supposition is that PLNRT, particularly to higher doses afforded by IMRT, will decrease pelvic failure rate in select patients. We aim to establish the impact of two different PLNRT doses on long term quality of life (QOL).

**Methods and materials:**

Prostate cancer patients (n = 428) recorded baseline scores using the Expanded Prostate Cancer Index Composite (EPIC), prior to definitive or post-prostatectomy RT. PLNRT, if given, was prescribed to 45 or 54 Gy at 1.8 Gy per fraction. New EPIC scores were recorded 20–36 months after radiotherapy. Absolute change in each domain subscale and summary score was recorded, along with if these changes met minimally important difference (MID) criteria. A separate multivariate analysis (MVA) was performed for each measure. Subsequent dosimetric analysis was performed.

**Results:**

Frequency of a MID decline was significantly greater with PLNRT to 54 Gy for urinary function, incontinence, and overall. No urinary decline was correlated with PLNRT to 45 Gy. PLNRT to 54 Gy was significant for decline in urinary function, bother, irritative, incontinence, and overall score in one or both MVA models while 45 Gy was not. Postoperative status was significant for decline in urinary function, incontinence, and overall. Amongst postoperative patients, there was significantly greater decline in urinary function score in the salvage setting. Neither 54 nor 45 Gy significantly affected bowel subscale or overall score decline.

**Conclusions:**

Using conventional fractionation, adding PLNRT to 54 Gy, but not 45 Gy, correlates with worse urinary QOL, with postoperative patients experiencing a steeper decline. PLNRT had no significant impact on bowel QOL with either dose.

## Highlights

• Pelvic nodal radiation to 54 Gy correlates with worse urinary quality of life.
• Pelvic nodal radiation to 45 Gy does not correlate with urinary quality of life.
• Post-operative radiation resulted in greater urinary quality of life decline.
• Pelvic nodal radiation did not correlate with bowel quality of life.

## Introduction

Concerns regarding toxicity and efficacy often preclude elective pelvic lymph node radiotherapy (PLNRT) in prostate cancer. For definitive radiotherapy (RT), early trials found minimal or no benefit from adding PLNRT [1], [2], [3]. Relevance of these studies is limited with modern dosing, and studies have found mixed results regarding PLNRT [4], [5], [6], [7]. Using modern techniques and a hypofractionated regimen, Murthy et al. showed significantly improved biological failure-free survival and disease-free survival in high risk, node-negative patients [8]. This randomized trial is one of few studies to use patient-reported outcome measures (PROMs) to compare quality of life (QOL) outcomes of patients who received IMRT with or without PLNRT [5], [6], [9], [10], [11]. Only one included the postoperative setting [9].

Completed randomized trials for RT following radical prostatectomy (RP) have been limited to the prostate fossa, preventing analysis of PLNRTs effect on outcomes [12], [13], [14]. Retrospective studies have presented conflicting results [15], [16], [17], [18], [19]. As in the definitive setting, the morbidity of adding PLNRT to the prostate fossa is unclear [16], [20], [21], [22], though it is offered by most physicians [23]. Findings from NRG Oncology/RTOG 0534 SPORRT seem to support this practice, demonstrating lower rates of progression with PLNRT [24].

The Expanded Prostate Cancer Index Composite (EPIC) is practical way to measure patient QOL [25], [26], [27]. Only two published studies have used the EPIC questionnaire to compare QOL outcomes by treatment with or without PLNRT [9], [10]. Each lacked statistical analysis by intra-individual change. Additionally, EPIC outcomes following radiation have not previously been analyzed by dosimetry, and no studies were found comparing QOL outcomes using PROMs by nodal dose. Our long-term supposition is that higher doses of PLNRT afforded by IMRT will decrease pelvic failure rate in high-intermediate and high-risk patients (28). We aim to establish the toxicity of increased pelvic nodal dose. We use EPIC questionnaires to analyze QOL changes in patients treated for prostate cancer in the definitive and postoperative settings, with or without PLNRT up to 54 Gy.

## Materials and methods

**Fig. 1:**
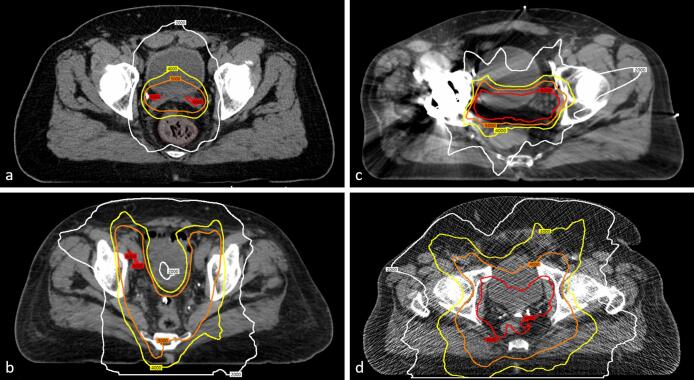
Four patients with 60 (red), 40 (orange), 30 (yellow), and 20 (white) Gy isodose lines visualized on the superiormost axial plane with 60 Gy isodose lines. The patients received: definitive radiation with a) 0 Gy to the nodes (20 cc prostate, 151 cc bladder) and b) 54 Gy to the nodes (22 cc prostate, 127 cc bladder); postoperative radiation with c) 0 Gy to the nodes (67 cc prostate fossa, 189 cc bladder) and d) 54 Gy to the nodes (41 cc prostate fossa, 189 cc bladder).

Between 2011 and 2017, 854 men were seen for consultation regarding prostate or prostate fossa RT. Institutional Review Board approval was obtained for prospective collection of QOL information using EPIC at consultation and follow-up[25]. All patients were treated without breaks using Novalis Tx (Brainlab, Munich, Germany) or Clinac® 2100C/D (Varian Medical Systems, Palo Alto, CA) linear accelerators with IMRT via volumetric modulated arc therapy or step-and-shoot in 1.8–2 Gy fractions (see Fig. 1). An initial goal was set to collect follow-up questionnaires at 23–25 months following RT at a clinical visit or via mail. However, timely collection proved difficult with limited resources, and with evidence for functional stability over time, we broadened inclusion criteria to 20–36 months following RT [6], [20], [28], [29].

Patients who underwent brachytherapy boost, additional salvage therapies, presented with distant metastatic disease, or did not complete a baseline and follow-up EPIC were excluded. Patients with intact prostate at the time of initial consultation who opted for radical prostatectomy were only included if they presented later for consideration of adjuvant/salvage radiation and completed a new baseline (post-surgery and pre-radiation) EPIC form. Androgen deprivation therapy (ADT) was given at physician’s discretion. When given, elective lymphatic coverage spanned craniocaudally from the bifurcation of the common iliac vessels to coccyx tip with perirectal and presacral coverage. The sexual domain was removed once it was noticed this improved return rates.

Absolute change in each domain summary and subscale score was recorded and categorized by whether this change met minimally important difference (MID) criteria developed by the PROST-QA Consortium [30]. These criteria are a score threshold constituting clinically relevant change in symptom burden for prostate cancer survivors. The urinary (GU) domain was comprised of the subscales function, bother, irritative, incontinence with a domain summary (overall) score. The bowel (GI) domain was comprised of the function and bother subscales with a domain summary (overall) score. The hormonal domain was comprised of the function and bother subscales with a domain summary (overall) score [25].

All baseline comparisons were done using bivariate analysis methods. Multivariate analysis (MVA) was performed using clinically relevant variables shown to be significant by Wilcoxon-rank-sum or Kruskal-Wallis tests. First, a generalized linear model was used to assess continuous value changes in each domain summary and sub-scale score. An additional MVA was performed with a proportional odds model to assess discrete score change using MID criteria (MID increase, MID decrease, or neither). Race, hormone status, and staging characteristics were not included in either MVA due to lack of significance by Wilcoxon-rank-sum or Kruskal-Wallis tests. P-value significance was set to <0.05. Variables included were age, RT setting (intact prostate/definitive or post prostatectomy), pre-treatment score, and nodal dose, year of RT, IMRT type (VMAT vs. step and shoot), and time interval from RT completion to EPIC follow-up.

Spearman rank tested for correlation between absolute domain summary or subscale score change and the percentage of organs at risk occupied by given isodose volumes. When a significant correlation was identified, isodose volume cut-points were developed based on receiver operating characteristic curve analyses maximizing sensitivity and specificity for MID decline. For each cut-point developed, areas under the curve (AUC) were developed and tested for comparative significance using omnibus and pairwise tests [31].

For comparative dosimetry by prescribed nodal dose, there was limited data available for patients treated to 45 Gy (n = 5) compared to 54 Gy (n = 180) and 0 Gy (n = 65). Using 54 Gy patients with available treatment plans, a simple random sampling was performed with observations clustered by post-op status, bladder V20, and small bowel V40 with a sampling rate of 0.1. Sixteen patients were selected to be dosimetrically representative of the 54 Gy population. Their original treatments plans were reoptimized to a nodal prescription dose of 45 Gy with an identical total dose and number of fractions to the prostate or prostate fossa after a subsequent sequential boost. Prior optimizer settings were scaled appropriately while maintaining prescription isodose coverage.

## Results

EPIC scores were recorded prior to and 20–36 months following IMRT in 428 of 523 eligible patients. See Table 1 for patient characteristics. At baseline prior to RT, post-operative patients had significantly worse baseline urinary function, incontinence, and overall score (all p-value < 0.0001) compared to definitive RT patients. There were no other significant differences.

**Table 1:** Patient Characteristics.

		Definitive RT	Postoperative RT	
			Adjuvant	Salvage
T stage*	1	218 (51)	0	0
	2	48 (11)	8 (2)	78 (31)
	3	2 (1)	36 (8)	38 (18)
N stage*	1	3 (1)**	6 (3)	12 (5)
Gleason†	≤6	44 (10)	0	46 (21)
	7	160 (37)	18 (4)	40 (51)
	≥8	64 (15)	26 (6)	30 (28)
Race	White	186 (43)	27 (6)	87 (70)
	Black	60 (14)	15 (4)	19 (22)
	Other	22 (5)	2 (1)	10 (8)
Nodal Dose (Gy)	0	126 (47)	3 (1)	2 (1)
	45	16 (6)	5 (1)	11 (3)
	54	125 (47)	36 (8)	103 (24)
Hormones††		71 (26)	6 (1)	16 (4)
PSA pre-RT median (range), ng/ml		7.8 (0.2–380)	0.0 (0.0–3.9)	0.4 (0.1–15.9)

Abbreviations: T, tumor; N, node; PSA, prostate specific antigen; RT, radiotherapy; Gy, gray.

*pathologic staging if postoperative and clinical if definitive.

**These three patients received an integrated boost to 60 Gy at 2 Gy per fraction for gross 1–2 cm lymph nodes.

†from surgical specimen if postoperative and biopsy if definitive.

††Immediately prior to, during, or after radiotherapy prior to follow-up.

Patients treated to 0, 45 and 54 Gy had MID decline frequencies as presented in Table 2. Compared to 0 Gy, the frequency of MID decline was significantly greater in patients treated to 54 Gy for urinary function (29% vs. 46%, p = 0.0024), urinary incontinence (35% vs. 53%, p = 0.0007), and urinary overall (30% vs. 42%, p = 0.0218). The respective frequencies of MID decline with 45 Gy were not significantly different. No other bowel or urinary scores in 45 or 54 Gy patients had significantly greater MID decline frequency. Divided by nodal dose and treatment setting, domain summary or subscale changes had no MID change or a MID improvement in 46–100% of patients (see Supplementary Table 1).

**Table 2:** Patients Experiencing a MID Decline by Nodal Dose.

		All Patients					Definitive Radiotherapy					Postoperative Radiotherapy				
Domain	Subscale/Summary Score	0 Gy (n = 131)	45 Gy (n = 33)	p-value*	54 Gy (n = 264)	p-value*	0 Gy (n = 126)	45 Gy (n = 16)	p-value*	54 Gy (n = 125)	p-value*	0 Gy (n = 5)	45 Gy (n = 16)	p-value*	54 Gy (n = 139)	p-value*
Urinary	Function	28.9%	25.8%	0.7308	45.5%	0.0024	30.2%	20.0%	0.5519	40.9%	0.0893	0.0%	31.3%	0.2776	49.6%	0.0591
	Bother	31.3%	36.4%	0.5781	39.8%	0.1004	32.5%	31.3%	0.9173	34.4%	0.7548	0.0%	41.2%	0.1348	44.6%	0.0702
	Irritative	23.5%	35.5%	0.1765	29.8%	0.2143	24.6%	26.7%	1.0000	23.0%	0.7836	0.0%	43.8%	0.1235	35.7%	0.1645
	Incontinence	35.1%	27.3%	0.3951	53.0%	0.0007	36.4%	18.8%	0.1604	51.6%	0.0148	0.0%	35.3%	0.2663	54.4%	0.0225
	Overall	29.8%	25.8%	0.6656	42.2%	0.0218	31.0%	20.0%	0.5510	34.5%	0.5749	0.0%	31.3%	0.2776	48.8%	0.0601
Bowel	Function	29.8%	32.3%	0.7864	32.9%	0.5424	31.0%	46.7%	0.2504	32.7%	0.7814	0.0%	18.8%	0.5489	33.1%	0.1774
	Bother	34.3%	27.3%	0.4398	38.0%	0.4704	35.7%	31.3%	0.7276	40.3%	0.4447	0.0%	23.5%	0.5352	36.0%	0.1635
	Overall	33.9%	29.0%	0.6079	37.5%	0.5000	35.3%	46.7%	0.3918	38.4%	0.6334	0.0%	12.5%	1.0000	36.7%	0.1609

Abbreviations: MID, minimally important difference; Gy, gray.

*compared to 0 Gy.

On subset analysis, the frequency of MID incontinence decline remained significantly greater with 54 Gy in definitive (36 vs. 52%, p = 0.0148) patients, with urinary function trending toward significance (30% vs. 41%, P = 0.0893). Only 5 postoperative patients received 0 Gy, with the frequency of MID incontinence decline remaining significantly greater with 54 Gy (0 vs. 54%, p = 0.0225).

Amongst postoperative patients, by Wilcoxon-rank-sum test, the salvage setting had greater absolute declines in urinary function score (p = 0.0254), with a trend toward greater decline in urinary incontinence (p = 0.0564) and overall (p = 0.0714) scores compared to the adjuvant patients. Prior to radiation, salvage patients did not have significantly higher urinary function (median 80, range (12–100) vs. 77 (27–100), p = 0.3869), incontinence (69 (0–100) vs. 65 (8–100), p = 0.5965), or overall (81 (22–100) vs. 78 (32–100), p = 0.6447) scores compared to adjuvant patients. Similarly, all other pre-RT EPIC scores between salvage and adjuvant patients showed no significant difference.

MVA for changes in urinary and bowel symptoms were performed with two models (absolute and MID, see Supplementary Table 2). Variables entering the models included age, pretreatment score, prior prostatectomy, nodal dose, year of RT, IMRT method, and EPIC follow-up interval. Pretreatment score was significant in all domains and subscales by either MVA model. Postoperative status was significant for decline in overall urination and function in both models while incontinence was only significant in the absolute model. PLNRT to 45 Gy had no significance. PLNRT to 54 Gy was significant for decline in urinary function, incontinence, and overall in both models. Decline of urinary bother and irritative subscales as well as bowel function were significant only in the MID model. Year of RT was significantly related to decline in of urinary function, bother, irritative, incontinence, and overall subscales as well as the bowel overall and bother subscales in the absolute model. In the MID model, year of RT was only significant for overall urinary decline. Age was significant for decline in bowel function in both models. IMRT method and EPIC follow-up interval had no significance. No significant associations were found in the hormone domain and its subscales.

### Dosimetric analysis

#### Genitourinary

No significant dosimetric relationships were found for urinary irritative change in definitive or postoperative patients. Urinary function change weakly negatively correlated with V20 in postop patients (cut-point for V20 of 100% with a PPV of 57%). No other significant relationships were found between dosimetric parameters and subscales postoperatively.

In definitive patients, V20, 30,40,50,60 and 70 all negatively correlated with urinary incontinence change. Isodose volume cut-points each showed a MID decline with positive predictive value (PPV) of > 44%: V70 of 9%, V60 of 16%, V50 of 31%, V40 of 50%, V30 of 71%, and V20 of 97%. A significant difference was detected between AUCs for these isodose volumes (p-value = 0.0132). The V20 cut point of 97% had the highest AUC (p-values all ≥ 0.13 by pairwise tests). V20 moderately correlated (cut-point for V20 of 98% with a PPV of 46%) with urinary function change while V30, 40, 50, and 60 weakly correlated (cut-point of 16–71% with a PPV of 39–46%). A significant difference between AUCs was not detected for these isodose volumes (p-value = 0.1612). Urinary bother changes weakly correlated with V30 (cut-point of 72% with a PPV of 32%).

**Fig. 2:**
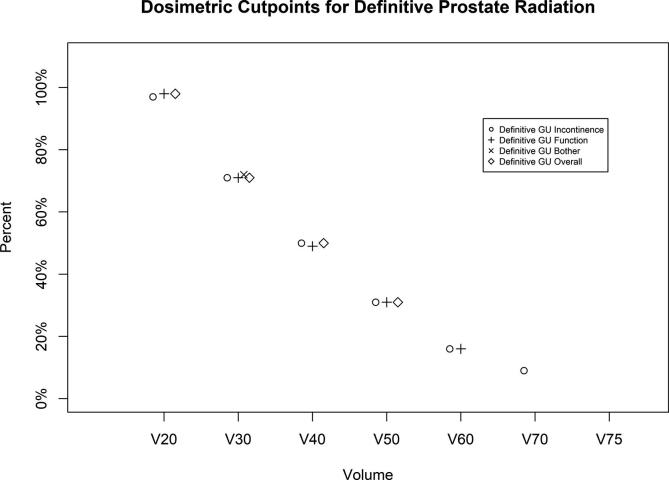
Bladder isodose volume cut-points developed for a minimally important difference decline in urinary subscale or overall score with definitive radiotherapy.

Urinary overall score change had significant correlations only in definitive patients (V20-50, cut-points of 31–98% with PPVs of 29–36%). A significant difference was detected between AUCs for these isodose volumes (p-value = 0.0359). The V20 cut point of 98% had the highest AUC and was significantly higher than V40, the lowest AUC (see Fig. 2 and Supplementary Table 3).

Using 16 dosimetrically representative 54 Gy patients, planning to 45 Gy would have resulted in a significantly decreased bladder V20 (median 93.5 [75–100 Gy], vs 98.0 [90–100 Gy], p = 0.0086). Bladder V30, V40, and V50 were decreased without reaching significance.

#### Gastrointestinal

No significant correlations were found for bowel function, bother, or overall change. A significant, weakly negative correlation was found for small bowel V40 and V45 and bowel bother change, with respective cut-points at 6% (37% PPV) and 3% (34% PPV). A significant difference between AUCs was not detected for these isodose volumes (p-value = 0.1376).

Per dosimetrically representative sampling, planning to 45 Gy would have resulted in a significantly decreased small bowel V40 (median 3 [0–18 Gy], vs 11 [1–54 Gy], p = 0.0058), V45 (median 0 [0–3 Gy], vs 5 [0–36 Gy], p < 0.0001), and V50 (median 0 [0–0 Gy], vs 1 [0–7 Gy], p = 0.0007) Small Bowel V52 (median 0 [0–0 Gy], vs 0 [0–2 Gy], p = 0.1639) was decreased without reaching significance. There were no significant differences in rectal V20-75.

## Discussion

PLNRT to 54 Gy significantly impacted urinary QOL compared to 0 Gy, while PLNRT to 45 Gy did not. Approximately 30% of all patients had MID changes in urinary function, with no significant differences between 0 and 45 Gy. For those receiving 54 Gy, there was a difference in MID changes for function and incontinence, contributing to an increased rate of MID overall urinary score decline by 11.2–16.4%. Noteworthy is that three of the five urinary function components are also measures of incontinence. Significant differences were driven by a minority of patients, as roughly one-fourth to half of patients had no MID changes or improvement in the various urinary measures. Overall, approximately 30% had MID changes in bowel function, but there was no difference between 0, 45 Gy or 54 Gy pelvic dose. Patients who had postoperative RT experienced a greater decline in urinary QOL than those treated in the definitive setting. Most available literature compared morbidity with or without PLNRT using Radiation Therapy Oncology Group (RTOG), European Organisation for Research and Treatment of Cancer (EORTC) or Common Terminology Criteria for Adverse Events (CTCAE) toxicity scales. Less literature exists comparing PROMs (see Table 3). We contextualize our findings using these studies.

**Table 3:** Literature Comparing Patient Reported Outcomes With and Without Pelvic Lymph Node Radiation.

Author	Year	Random-ized?	Setting	Modality	patients (n)	prostate dose total (Gy)	prostate dose per fraction (Gy)	nodal dose total (Gy)	nodal dose per fraction (Gy)	Hormones (%)	PROM	Significant QOL changes with PLNRT:	Follow-up notes
Hanlon	2001	No	Definitive	3DCRT	139	64–78	2.1*	46–50	2.1*	0	AUA SPI, BPH II, bowel/bladder functioning surveys	Increased bowel pad use, rectal urgency, nocturia, urinary bother, worse bowel functioning satisfaction	Median 54 months
Pommier	2007	Yes	Definitive	3DCRT	444	66–72/ 65.25	1.8–2/2.25	46–46.8/ 45	1.8–2/2.25	58.5	EORTC QLQ-C30, IPSS, Sexual Function Index	None	12 and 24 months
Melotek	2015	No	Postoperative	IMRT	33–102	66–68/ 66.6–68.4	1.8	50.4	1.8	56	EPIC-26	None for bowel/sexual function (all timepoints), worse urinary continence (baseline to 24 months), irritation or obstruction (2 months only)	87% salvage, group comparisons at baseline, 2,6,12,18,24,36,48 months
Lilleby	2016	No	Definitive	IMRT†	206	74	–	50	–	100	UCLA-PCI, SF-12, FQ	Increased fatigue and anxiety, worse bowel bother at 12 months and function at 36 months (no difference at baseline)	Urinary function and bother worse at all timepoints (baseline,12, 24, 36 months)
Dearnaley	2018	Yes	Definitive	IMRT	124	74	2	55–60	1.49–1.62	100	IBDQ, VIQ, IPSS	Urinary and bowel outcomes roughly similar (no reported P values)	2.5, 4.5, 6, 12, 18, and 24 months
Murthy	2020	Yes	Definitive	IMRT	224	68	2.72	50	2	99–100	EORTC QLQ-C30 and PR-25	None	Every 3–6 months post RT
Parry	2020	No	Definitive	IMRT	5468	74 (median)	2	–	–	79.8	EPIC-26, EQ-5D-5L	Worse sexual function score considered not clinically relevant	Mailed at least 18 months after diagnosis, baseline scores for comparison

Abbreviations: PSA, prostate specific antigen; RT, radiotherapy; PROM, patient reported outcome measures; QOL, quality-of-life; PLNRT, pelvic lymph node radiotherapy; AUA SPI, American Urological Association Symptom Problem Index; BPH II, Benign Prostatic Hyperplasia Impact Index; EORTC QLQ-C30, European Organization for the Research and Treatment of Cancer Core Quality of Life Questionnaire; IPSS, International Prostate Symptom Score questionnaire; EPIC-26, Expanded Prostate Cancer Index Composite; UCLA-PCI, University of California, Los Angeles - Prostate Cancer Index; FQ, Fatigue Questionnaire; SF-12, Short Form 12 questionnaire; IBDQ, Irritable Bowel Disease Questionnaire; VIQ, Vaizey Incontinence Questionnaire; EORTC QLQ-PR25, European Organization for the Research and Treatment of Cancer Quality of Life Questionnaire - Prostate Cancer Module; EQ-5D-5L, EuroQol Group 5 dimension 5 level questionnaire.

*Prescribed to ICRU reference point.

^†^with 3DCRT boost.

### Definitive prostate radiotherapy

Of three randomized studies using three-dimensional conformal radiotherapy (3DCRT) in the PSA era, only RTOG 94-13 identified any significant toxicity increase with PLNRT, isolated to G ≥ 3 GI side effects and G ≥ 3 lymphopenia in the neoadjuvant ADT subgroups [3], [32], [33]. A significant concern regarding physician toxicity reporting is susceptibility to underascertainment [34]. Hanlon et al. found that despite overall patient satisfaction, those who received PLNRT had decreased bowel functioning satisfaction, increased use of anal pads, worse nocturia and urinary bother [35]. However, using different PROMs, GETUG 01 reported no significant difference in overall QOL, urinary, or sexual function with PLNRT at 12 or 24 months [3].

Increased use of PROMs and IMRT standardization has yet to clearly reveal PLNRT morbidity. Published results from the PIVOTAL study without p-values indicated higher G ≥ 2 acute GI toxicity rates and higher IBDQ scores with PLNRT [5]. However, as with other cohorts, GU toxicity and PROMs were similar [4], [7], [36]. The POP-RT study found only G ≥ 2 late GU toxicity significantly increased without any significant differences by PROMs [6]. Our findings indicate nodal dose may play a significant role in in GU PROMs. While PLNRT to 45 Gy was not associated with greater decline in any EPIC GU scores, 54 Gy was. Simple statistics describing the likelihood of MID decline may be the most informative at a clinical level. In all patients, 54 Gy PLNRT significantly increased the likelihood of a meaningful decline in urinary function, incontinence, and overall. Multiple MVA models supported this finding. On analysis of exclusively definitive patients, only incontinence remained significant. There was no signal that 45 Gy PLNRT increased likelihood of meaningful decline. Thus, from a urinary QOL standpoint the cost of PLNRT appears to be minimal, with a small but significant increase in morbidity escalating PLNRT from 45 to 54 Gy. Bowel QOL did not appear to be significantly affected by PLNRT, even to 4 Gy.

Early risk analysis of PLNRT for definitive prostate cancer RT showed significant increases in low to moderate dose regions of the rectum and bladder without associated increase in late normal tissue complication probability (NTCP) [37]. A small cohort found significantly increased bladder parameters with PLNRT, particularly V20, were not associated with G ≥ 2 GU toxicity. Similarly, rectal and bowel parameters increased significantly with PLNRT were not associated with acute or late GI toxicity [7] More recent studies have corroborated these reports [4], [6].

Amongst all patients, we found several relationships indicating low to moderate isodose volumes, particularly V20, to high percentages of the bladder were predictive of GU EPIC score decline. Replanning a representative sampling of 54 Gy patients to 45 Gy resulted in decreasing size of low to moderate bladder isodose volumes (V20-V50). This difference may explain why PLNRT to 54 Gy resulted in significant GU QOL decline while PLNRT to 45 Gy did not. Generated cut-points were fairly consistent across urinary subscales and overall score. Differences in small bowel dose had no significant impact on bowel QOL.

### Postoperative radiotherapy

Postoperatively, acute and late GU and GI toxicity have each been reported as similar or worse with PLNRT in non-randomized cohorts. Results of SSPORT indicate significant increases in toxicity with PLNRT are mainly hematologic [38]. With regard to PROMs, Melotek et al. found similar sexual and GI function with PLNRT. Urinary continence was worse at 24 months but it was also worse at baseline. Significantly worse irritation and obstruction dissipated after 2 months [9]. Parry et al. found no significant differences using EPIC-26 or EQ-5D-5L at ≥ 18 months with the exception of sexual function score, which was not clinically meaningful. Baseline scores were not available for comparison [10], [39].

In this study, although postoperative patients had significantly worse baseline scores for urinary function, incontinence, and overall score, postoperative status was associated with a significantly greater decline on MVA. There may be a lower tolerance for radiation following surgery. Adjuvant RT has previously demonstrated increased GU side effects compared to salvage RT [20], [40]. We found that patients reported a greater decline of urinary function, incontinence, and overall scores in the salvage setting. Lower bowel or urinary functioning closer to surgery, and hence bias toward less dramatic score drops after RT in the adjuvant setting, was thought to be a possible reason for this unexpected result. However, further investigation showed no significant difference between any of the urinary or bowel scores in adjuvant vs. salvage patients. Thus, the reason for this finding is unclear, and could simply be a consequence of imbalanced patient distribution (73% salvage vs. 28% adjuvant). If not, there may be an association between the chronology or impetus behind post-operative radiation and PROMs over time that has not typically been demonstrated with physician reported urinary toxicity.

Only 5 patients did not receive PLNRT postoperatively, and of those who did, only 10.3% received 45 Gy. Thus, no firm conclusions can be made with this data regarding the associations of PLNRT and urinary QOL in this population. That being said, PLNRT to 54 Gy resulted in a small but significantly increased frequency of meaningful urinary decline, while 45 Gy did not.

There was some signal for v40 and v45 affecting bowel bother in postoperative patients, but this did not translate into affecting either the overall score or the likelihood of a meaningful decline in bowel bother with PLNRT. Similarly, only Bladder V20 was associated with a decline of urinary function. Unlike definitively treated patients, bladder isodose volumes were similar with either PLNRT dose. With more severe decline in urinary QOL following RT one might expect a stronger signal of dosimetric measures on QOL as well. However, a greater magnitude of decline may lead to less variation in urinary QOL changes. Further, because the bladder is part of the target volume when treating the prostate fossa, dosimetric variation may be decreased, and altering treatment may be less impactful on urinary QOL and more difficult.

The main limitation of this study is the underrepresentation of postoperative patients who did not receive PLNRT and patients who received PLNRT to 45 Gy. Other study limitations include lack of randomization and potential participation bias with regard to completion of follow-up EPIC forms. Though follow-up times were not found to significantly affect any EPIC changes, post RT forms were collected once within a relatively wide time period that would not capture or allow for comparison of acute and sub-acute toxicities. Complete treatment plans were unavailable for some patients, resulting in considerably limited and underpowered dosimetric analysis.

## Conclusion

Using conventional fractionation, PLNRT to 54 Gy, but not 45 Gy, correlates with worse urinary QOL when compared to no pelvic radiation. This difference may be due to significantly increased low to moderate bladder isodose volumes. Despite lower baseline scores, postoperative patients have a steeper decline in urinary QOL following RT, particularly in the salvage setting. PLNRT has minimal impact on bowel QOL. Optimal dosing of PLNRT in prostate cancer requires additional studies carefully assessing control and morbidity, using both physician and patient reported outcomes.

## Funding

None.

## Declaration of Competing Interest

The authors declare that they have no known competing financial interests or personal relationships that could have appeared to influence the work reported in this paper.
