# Supplementary material for: The cost of elective nodal coverage in prostate cancer: Late quality of life outcomes and dosimetric analysis with 0, 45 or 54 Gy to the pelvis
Source: Clin Transl Radiat Oncol. 2022 Jun 27;36:63–9. doi: 10.1016/j.ctro.2022.06.008 (PMC9256976; doi:10.1016/j.ctro.2022.06.008)
Supplement: Supplementary data 1 [file mmc1.docx]

| Supplementary Table 1: Meaningfully Important Differences in Urination by EPIC Measures Following Radiation | | | | | | | | | | |
| --- | --- | --- | --- | --- | --- | --- | --- | --- | --- | --- |
| EPIC Category | Treatment Setting | | | | | | | | | Nodal Dose (Gy) |
|  | Any (%) | | | Definitive (%) | | | Postoperative (%) | | |  |
|  | Improved | None | Declined | Improved | None | Declined | Improved | None | Declined |  |
| Urinary Function | 22 | 49 | 29 | 20 | 50 | 30 | 80 | 20 | 0 | 0 |
|  | 19 | 55 | 26 | 13 | 67 | 20 | 25 | 44 | 31 | 45 |
|  | 16 | 43 | 39 | 17 | 43 | 41 | 16 | 35 | 50 | 54 |
|  | 18 | 43 | 39 | 18 | 48 | 35 | 19 | 35 | 46 | Any |
| Urinary Bother | 36 | 33 | 31 | 34 | 33 | 33 | 80 | 20 | 0 | 0 |
|  | 24 | 39 | 36 | 31 | 38 | 31 | 18 | 41 | 41 | 45 |
|  | 25 | 35 | 40 | 28 | 38 | 34 | 23 | 32 | 45 | 54 |
|  | 29 | 35 | 37 | 31 | 36 | 33 | 24 | 33 | 43 | Any |
| Urinary Irritation | 34 | 43 | 24 | 32 | 44 | 25 | 80 | 20 | 0 | 0 |
|  | 19 | 45 | 36 | 20 | 53 | 27 | 19 | 38 | 44 | 45 |
|  | 27 | 43 | 30 | 30 | 47 | 23 | 25 | 40 | 36 | 54 |
|  | 29 | 43 | 28 | 30 | 46 | 24 | 26 | 39 | 35 | Any |
| Urinary Incontinence | 26 | 39 | 35 | 25 | 39 | 36 | 60 | 40 | 0 | 0 |
|  | 24 | 49 | 27 | 19 | 63 | 34 | 29 | 35 | 35 | 45 |
|  | 18 | 29 | 53 | 14 | 34 | 52 | 21 | 25 | 54 | 54 |
|  | 21 | 34 | 46 | 20 | 38 | 42 | 23 | 26 | 51 | Any |
| Urinary Overall | 31 | 40 | 30 | 29 | 41 | 31 | 80 | 20 | 0 | 0 |
|  | 19 | 55 | 26 | 27 | 53 | 20 | 13 | 56 | 31 | 45 |
|  | 20 | 38 | 42 | 21 | 44 | 35 | 19 | 33 | 49 | 54 |
|  | 23 | 40 | 37 | 25 | 43 | 32 | 20 | 35 | 45 | Any |

| Supplementary Table 2: Multivariate Analyses | | | | | | | | | | |
| --- | --- | --- | --- | --- | --- | --- | --- | --- | --- | --- |
|  |  | MVA model | Age: 1-year increase | Pre-treatment Score: 1-point increase | Post op: Yes vs No | Node Dose: 45 vs 0 | Node Dose: 54 vs 0 | Year of RT: 1-year increase | Step and Shoot*: Yes vs No | EPIC follow-up: 1-year increase |
| Urinary | Function | Absolute | 0.8453 | <0.0001 | 0.0012 | 0.95 | 0.0334 | 0.0277 | 0.7452 | 0.898 |
|  |  | MID | 0.9542 | <0.0001 | 0.0267 | 0.9252 | 0.0003 | 0.0743 | 0.9917 | 0.6957 |
|  | Bother | Absolute | 0.7467 | <0.0001 | 0.4359 | 0.4653 | 0.0933 | 0.0107 | 0.6277 | 0.3632 |
|  |  | MID | 0.8424 | <0.0001 | 0.1349 | 0.5159 | 0.0015 | 0.0647 | 0.4219 | 0.2135 |
|  | Irritative | Absolute | 0.8977 | <0.0001 | 0.9474 | 0.6603 | 0.0959 | 0.0248 | 0.3324 | 0.8949 |
|  |  | MID | 0.4652 | <0.0001 | 0.3344 | 0.579 | 0.0101 | 0.4619 | 0.1579 | 0.175 |
|  | Incontinence | Absolute | 0.7757 | <0.0001 | 0.046 | 0.5881 | 0.0492 | 0.0468 | 0.5795 | 0.4617 |
|  |  | MID | 0.9673 | <0.0001 | 0.2164 | 0.6938 | <0.0001 | 0.0525 | 0.8028 | 0.9439 |
|  | Overall | Absolute | 0.9968 | <0.0001 | 0.0182 | 0.7355 | 0.0367 | 0.0072 | 0.7849 | 0.8023 |
|  |  | MID | 0.9797 | <0.0001 | 0.0045 | 0.8059 | 0.0002 | 0.0077 | 0.7342 | 0.7126 |
| Bowel | Function | Absolute | 0.0436 | <0.0001 | 0.7653 | 0.871 | 0.3036 | 0.1117 | 0.7617 | 0.1575 |
|  |  | MID | 0.0458 | <0.0001 | 0.3622 | 0.7742 | 0.0083 | 0.2847 | 0.2647 | 0.2861 |
|  | Bother | Absolute | 0.9799 | <0.0001 | 0.6065 | 0.8133 | 0.1016 | 0.0329 | 0.9174 | 0.3272 |
|  |  | MID | 0.147 | <0.0001 | 0.3316 | 0.9823 | 0.0781 | 0.3937 | 0.5133 | 0.8148 |
|  | Overall | Absolute | 0.2609 | <0.0001 | 0.5722 | 0.985 | 0.1954 | 0.0447 | 0.982 | 0.1633 |
|  |  | MID | 0.0912 | <0.0001 | 0.2679 | 0.9026 | 0.063 | 0.3105 | 0.3115 | 0.1334 |
| Abbreviations: MVA: multivariate analysis; RT, radiotherapy; EPIC, Expanded Prostate Cancer Index Composite | | | | | | | | | | |
| *Patients all received Intensity Modulated Radiotherapy via either 'Step and Shoot' or 'arc.' | | | | | | | | | | |

| Supplementary Table 3: Urinary EPIC Score Changes by Bladder Dosimetry | | | | | | | | | | | |
| --- | --- | --- | --- | --- | --- | --- | --- | --- | --- | --- | --- |
| Setting | Bladder Volume (v in %) at Dose (Gy) | Function* | p-value | Bother* | p-value | Irritative* | p-value | Incontinence* | p-value | Overall* | p-value |
| All | v75 | 0.14685 | 0.0321 | 0.11531 | 0.071 | 0.16326 | 0.0175 | 0.0779 | 0.2218 | 0.16485 | 0.0159 |
|  | v70 | -0.10592 | 0.1234 | -0.03528 | 0.5822 | 0.03756 | 0.5879 | -0.10734 | 0.0917 | -0.07512 | 0.2754 |
|  | v60 | -0.19683 | 0.0039 | -0.07596 | 0.2355 | 0.00163 | 0.9813 | -0.17474 | 0.0057 | -0.1507 | 0.0278 |
|  | v50 | -0.23562 | 0.0005 | -0.11447 | 0.0731 | -0.02415 | 0.7276 | -0.22393 | 0.0004 | -0.1927 | 0.0047 |
|  | v40 | -0.2522 | 0.0002 | -0.14307 | 0.0247 | -0.04634 | 0.5037 | -0.24866 | <.0001 | -0.20991 | 0.002 |
|  | v30 | -0.26136 | 0.0001 | -0.16277 | 0.0105 | -0.06992 | 0.3125 | -0.26108 | <.0001 | -0.22188 | 0.0011 |
|  | v20 | -0.31993 | <.0001 | -0.15759 | 0.0132 | -0.08414 | 0.2238 | -0.28921 | <.0001 | -0.25188 | 0.0002 |
| Definitive | v75 | -0.01253 | 0.8868 | 0.02128 | 0.7937 | 0.10927 | 0.2163 | -0.0571 | 0.4795 | 0.01627 | 0.8534 |
|  | v70 | -0.12653 | 0.1485 | -0.03683 | 0.6507 | 0.05428 | 0.5403 | -0.16455 | 0.04 | -0.08334 | 0.3428 |
|  | v60 | -0.20181 | 0.0201 | -0.0432 | 0.5953 | 0.0868 | 0.3267 | -0.22515 | 0.0046 | -0.12657 | 0.1484 |
|  | v50 | -0.24937 | 0.0038 | -0.08582 | 0.2904 | 0.05672 | 0.5222 | -0.29016 | 0.0002 | -0.1721 | 0.0483 |
|  | v40 | -0.27382 | 0.0014 | -0.14087 | 0.0814 | 0.000261 | 0.9977 | -0.32017 | <0.0001 | -0.20358 | 0.019 |
|  | v30 | -0.25999 | 0.0025 | -0.16684 | 0.0385 | -0.04132 | 0.6412 | -0.31637 | <0.0001 | -0.21406 | 0.0135 |
|  | v20 | -0.3035 | 0.0004 | -0.14372 | 0.0753 | -0.03981 | 0.6535 | -0.34752 | <0.0001 | -0.22265 | 0.0101 |
| Postop | v75 | 0.0611 | 0.589 | 0.08085 | 0.4447 | 0.09415 | 0.4043 | 0.03579 | 0.7356 | 0.08717 | 0.4402 |
|  | v70 | 0.04405 | 0.6971 | 0.04537 | 0.6684 | 0.09856 | 0.3825 | 0.01614 | 0.879 | 0.0627 | 0.5793 |
|  | v60 | -0.00753 | 0.947 | -0.0193 | 0.8555 | 0.02028 | 0.8578 | -0.02253 | 0.8317 | -0.00314 | 0.9779 |
|  | v50 | -0.06703 | 0.5533 | -0.06481 | 0.5404 | -0.03041 | 0.7882 | -0.05509 | 0.6029 | -0.06658 | 0.5559 |
|  | v40 | -0.08811 | 0.4353 | -0.04689 | 0.658 | -0.02808 | 0.8041 | -0.07886 | 0.456 | -0.07519 | 0.5059 |
|  | v30 | -0.14584 | 0.1945 | -0.0474 | 0.6545 | -0.03281 | 0.7719 | -0.09463 | 0.3706 | -0.09968 | 0.3771 |
|  | v20 | -0.25103 | 0.0235 | -0.09719 | 0.3577 | -0.0864 | 0.4443 | -0.1044 | 0.3229 | -0.19066 | 0.0882 |
| *Spearman Rank Correlation | | | | | | | | | | | |
